# The physical literacy promoting university: an organisational conceptual framework

**DOI:** 10.3389/fspor.2026.1809210

**Published:** 2026-07-02

**Authors:** Leonie Schnith, Johannes Carl, Jochen Mayer

**Affiliations:** 1 Department of Sports Science, Institute of Health Sciences, University of Education Schwäbisch Gmünd, Schwäbisch Gmünd, Germany; 2 School of Health and Social Development, Institute for Physical Activity and Nutrition, Deakin University, Geelong, VIC, Australia

**Keywords:** health literacy, health-promoting university, organisational physical literacy, organisational structures, physical literacy, systems theory, tertiary education

## Abstract

Universities represent significant settings for shaping lifelong health- and physical activity-related behaviours and predispositions among their members. Existing research on physical literacy (PL), a multi-dimensional concept supporting an active and healthy lifestyle across the lifespan, has primarily focused on individual-based interventions rather than addressing the organisational context for PL promotion in universities. Therefore, the goal of the present article is to introduce the framework of the *Physical Literacy Promoting University (PLPU)* for embedding and fostering PL within the university setting from an organisational perspective. The framework was inspired by and structured according to an iterative, theory-guided conceptual framework development process, combining literature on PL promotion in university settings, organisational health literacy, and sociological systems theory. The organisational theoretical part of systems theory served as the overarching foundation, conceptualising universities as decision-based organisations. Building on these insights, central objectives and organisational components of a PLPU were identified through an iterative reflection and collaborative process. Universities can facilitate the systematic and sustainable integration of PL into their structures and processes by addressing five key components and their respective objectives: (1) recognising all members as dual actors in fostering and embedding PL; (2) aligning decision programmes, structures, and resources to embed and sustain PL; (3) ensuring coherent PL-related communication, decision-making, and task allocation; (4) establishing a PL-supportive campus culture; and (5) observing environmental developments and building relations with external stakeholders. By introducing the PLPU framework, this paper provides a foundation for settings-based public health strategies to integrate and foster PL within universities.

## 1 Introduction

Physical literacy (PL) has gained increasing research attention in recent years (1). Considered a person-related disposition, PL is understood as a holistic concept that addresses the physical, affective, cognitive, and social prerequisites (often bundled under the so-called “domains”) of an individual (2, 3). By contributing to physical activity (PA) behaviour and health (4), PL is considered a key determinant for a lifelong active and healthy lifestyle (5). Recent studies have highlighted the role and relevance of PL in the context of universities, as it shows considerable potential for supporting students in fostering long-term active health-oriented habits and dispositions (6, 7). This potential is particularly important given that the university phase is often accompanied by significant transitions, such as leaving home, gaining autonomy, and establishing new daily routines, which can challenge the health behaviours of students (8, 9). Approximately one-third of young adults are university students (10), and 594 million people worldwide are predicted to be enrolled in study programmes by 2040 (11). However, about 40% of university students do not meet the recommended PA guidelines (12, 13) and spend on average 9.28 h per day in a sedentary position (14). Thus, students are considered more sedentary and inactive compared to the general young adult population (14). Similar inactive and sedentary patterns have also been found among university staff members, as they spend large parts of their working day in a sitting position (15). Such an inactive lifestyle is associated with an increased risk of developing biopsychosocial health issues over the lifespan and a higher risk of morbidity and mortality, including non-communicable diseases like type 2 diabetes, cardiovascular diseases and cancer (16, 17).

To combat this public health issue, the World Health Organization (18) suggested in its Global Action Plan on Physical Activity 2018–2030 (GAPPA) that efforts should target not only individual behaviours but also create active environments and systems that foster and support active healthy lifestyles. While traditional PA interventions primarily focus on measurable individual behaviour, they tend to frame PA as an outcome influenced at the individual level and therefore only partially address the broader environmental conditions that shape opportunities for movement (19, 20). In contrast, PL adopts a broader and multidimensional perspective by acknowledging the interaction between individuals and their environment (21). In this context, promoting PL has been declared a key strategy for achieving the GAPPA goals (18), as it addresses both behavioural outcomes and the underlying person-environment interactions that shape lifelong engagement in physical activities.

### 1.1 Physical literacy as a holistic concept

PL is most commonly defined as “the motivation, confidence, physical competence, knowledge and understanding to value and take responsibility for engaging in physical activities for life” (2). Existing evidence demonstrates a substantial association between PL and actual engagement in physical activities (21, 22). PL embodies, through its holistic approach, a dynamic, non-linear journey throughout life, which can fluctuate depending on lived experiences, biographical changes, and individual perceptions (23, 24). This understanding is grounded in three philosophical tenets: monism emphasises the inseparability of body and mind as a coherent unit; phenomenology acknowledges the uniqueness of lived experiences, which can shape an individual's perception and reactions within future movement-related situations; and existentialism refers to the interaction with the environment through which individuals create their own identity and physical self-concept (5, 25, 26).

The concept of PL is associated with ongoing conceptual tensions (5). Although existing definitions generally share core characteristics, they partly differ in their focus, such as the inclusion of PL's philosophical underpinnings. This may contribute to a fragmented understanding of the concept. In addition, current assessment instruments predominantly rely on standardised quantitative approaches, often emphasising the physical and cognitive domains while neglecting the holistic, unique, and dynamic nature of PL (5, 27). This creates the risk of reducing PL to a set of measurable standardised competencies (e.g., motor skills), while neglecting an individual's embodied relationship with movement and its environmental context (28).

### 1.2 Physical literacy in universities

Although research on PL in university settings remains limited (29, 30), existing cross-sectional studies have shown positive associations of PL with physical fitness, mental health, and PA enjoyment among students (31–33). The few intervention-based approaches can mainly be grouped into curricular interventions within study programmes (primarily physical education courses), extracurricular movement programmes on campus, transition-focused programmes for first-year students, and lecturer education measures. Student-focused interventions have implemented a variety of traditional or non-traditional movement activities (e.g., “capture the flag” or HIIT sessions), primarily focusing on the physical domain (6, 7, 34, 35). Evidence also shows that pre-service teachers partly lack the skills to adequately educate students about PL in physical education (36, 37). Besides students, Choi et al. engaged lecturers in a professional development programme to enhance their theoretical and practical PL knowledge (35). Overall, these interventions have yielded outcomes regarding PA levels and/or participation and perceived PL.

However, the current evidence remains methodologically limited due to small sample sizes, short intervention durations, predominantly pilot or quasi-experimental designs, and a focus on self-report and varying assessment instruments. In addition, most interventions primarily target students, followed by lecturers. Administrative and auxiliary staff within universities remain neglected.

Despite the promising results of fostering PL (30, 38), the few existing interventions across universities also demonstrate some conceptual and practical shortcomings: they often adopt a relatively narrow focus on the physical domain. Additionally, limited consideration is given to the environmental conditions of universities, particularly how they shape the lived experiences and physical self-concept of their members in line with the philosophies of existentialism and phenomenology. This includes a limited acknowledgment of the interdependence between the individual and their environment (29, 30), as highlighted by socio-ecological models, which stress that an individual's behaviour and disposition are inevitably embedded within their social, cultural, and organisational environment (39, 40). This further supports the World Health Organization's (18) recommendation of creating active environments and systems for sustainably fostering PL and a lifelong active and healthy lifestyle. However, universities cannot be reduced to merely educational environments; they are complex organisations whose specific structures and processes shape the routines, behaviours, and dispositions of students and staff members (41).

Adopting an organisational perspective enables a more nuanced understanding of how universities function, respond to external demands, and shape internal structures and processes (39, 40). Most importantly, it highlights how they can serve (or not) to nurture the PL of members.

### 1.3 Aim and research question

While the potential of promoting PL at the individual level among university members has been acknowledged (6, 34), there is currently no framework that conceptualises how PL can be holistically embedded within the organisational structures of a university. The present article addresses this research gap by introducing the conceptual framework of the *Physical Literacy Promoting University* (PLPU). The key characteristics of this framework are introduced and outlined, guided by the following research question: Which components characterise the Physical Literacy Promoting University?

The article introduces and conceptually differentiates between personal and organisational PL, while also critically transferring ideas from organisational health literacy to the university context. These aspects form the framework's foundation for the systematic conceptualisation of embedding PL into the university setting. The goal of the framework is to provide theoretical and practical insights for practitioners and researchers in the university context. Moreover, it serves as a pathway for universities to systematically promote PL and foster an active, healthy lifestyle for students and staff members.

## 2 Development process of the conceptual framework

The development process was inspired by and structured around an iterative, theory-guided conceptual framework development process (42).

First, literature on PL promotion in universities, such as empirical interventions (6, 7, 34, 35), cross-sectional studies (31–33), and reviews (29, 30) were analysed to gain an in-depth understanding of existing approaches and their key characteristics (e.g., the predominant focus on individual behaviour). Second, literature on organisational health literacy (HL) (49, 50) and related conceptual frameworks were examined to identify organisational (49–52) principles and structural elements relevant for embedding HL in organisational contexts. Third, different organisational theoretical approaches were critically reflected upon regarding their suitability for conceptualising universities as organisations (43–45). Within this process, the organisational theoretical perspective of sociological systems theory emerged as the most suitable theoretical foundation. This is particularly due to its focus on organisations as decision-based and communication-driven systems, its explicit conceptualisation of organisational structures and processes, and its ability to analyse the interaction between organisations and their environment (46, 47).

In the fourth and final step, the framework was developed through an iterative process of synthesising, comparing, and integrating insights from the literature base using the systems theoretical perspective as the central foundation. The framework's key components were primarily derived from the central systems-theoretical considerations, particularly organisational roles and membership structures, decision-making premises, communication processes, and the relationship between organisations and their environment (46, 47). Throughout this highly collaborative process among the authors, continuous reflection was also undertaken to ensure alignment with the holistic PL concept.

Cognizant of the conceptual tensions regarding the PL concept (5), the framework seeks to avoid reductionist interpretations of PL. Besides focusing on increasing opportunities for movement-related experiences, it aspires to focus on organisational conditions that support motivation, confidence, personal meaning, inclusion, and long-term responsibility for engagement in PA and movement-related dispositions. In addition, the article introduces and conceptually differentiates between personal and organisational PL, while also critically transferring ideas from organisational health literacy to the university context. These aspects form the framework's foundation for the systematic conceptualisation of embedding PL into the university setting.

## 3 Theoretical background

### 3.1 Personal and organisational physical literacy

Although universities can be considered organisations (41, 46), the organisational perspective has received limited consideration within PL research. In comparison, HL, a concept with parallels to PL, has increasingly integrated such an organisational approach by differentiating between personal and organisational HL (49, 50).

*Personal HL* refers to an individual's ability to obtain, process, understand, and apply health information (50). In contrast, *organisational HL* demonstrates an organisation's capacity to create a HL-enhancing environment that supports individuals in navigating and using health-related information to foster their HL (49). Organisational HL, which emerged in the early 2000s, was underpinned by various disciplines such as organisational behaviour, healthcare management, implementation science, and quality improvement (49). Due to variations regarding goals and application areas, for instance, in healthcare organisations (51) or schools (52), different frameworks were developed to operationalise organisational HL (49).

Building on this distinction, we propose a similar differentiation for PL: *personal PL* and *organisational PL*, which, to the best of the authors' knowledge, have not been explicitly conceptualized. *Personal PL* focuses on an individual's abilities, understanding, confidence, and disposition to engage in PA throughout life by enhancing not only their physical but also their cognitive, social, and affective prerequisites. *Organisational PL* refers to an organisation's dynamic capacity to create, coordinate, communicate, and evaluate structures and processes that support the holistic development of its members' PL. This includes fostering various organisational conditions that address the physical, cognitive, social, and affective domains of PL. Accordingly, organisational PL shifts the perspective from solely focusing on individual dispositions towards the organisational capacities and conditions that enable and promote meaningful movement-related experiences. In this regard, organisational PL extends beyond an organisation that solely promotes PL.

While organisational HL focuses on enabling individuals to access, understand, and use health-related information within organisational contexts (49), PL extends beyond informational aspects (53) and should not be considered as a direct transfer from organisational HL. It additionally addresses embodied movement experiences, motivation, confidence, personal meaning, and the interaction between individuals and their environment. The idea of organisational PL is related to broader approaches such as settings-based health promotion and health-promoting universities. Settings-based health promotion emphasises the importance of supportive environmental conditions for fostering health within various settings (54). Building on that, health-promoting universities focus on improving health and wellbeing by concentrating on universities' environmental and contextual conditions (55). Organisational PL draws on these perspectives but places a strong focus on organisations and their specific structures, processes, capacities, and organisational conditions that support the long-term development of PL and its relation to lifelong active living.

### 3.2 Universities as organisations: a system-theoretical perspective

The conceptualisation of organisational PL serves as the basis for the PLPU framework. However, embedding PL in universities requires a coherent operationalisation and conceptualisation based on organisational theory. In comparison to other models and theories, such as institutional theory (45), socio-ecological models (39), and organisational change theory (43), systems theory was considered the most suitable foundation for the PLPU framework. While these approaches also provide valuable theoretical perspectives, systems theory particularly conceptualises organisations as self-referential systems that exist, reproduce, and further develop through internal decision-making and communication processes. It also provides structural components, which were considered highly relevant for understanding how organisations function (46, 47) and how PL can be structurally embedded within university structures.

Based on systems theory, universities typically pursue the purposes of (a) providing high-quality education, (b) generating knowledge through research, and (c) ensuring smooth administrative operations (41, 48). To accomplish these fundamental tasks, universities rely on communication and decision processes as central organisational functions, while being settings in which individuals work, learn, and interact with each other (41, 47, 48).

Against this background, universities are conceptualised as unique social systems characterised by their internal structures and the central role of communication and decision-making processes (56). This forms the foundation of a university's existence, reproduction, and further development. From this perspective, universities are considered self-contained and autopoietic organisations embedded in the educational system (54). One of the key principles of systems theory is the assumption that organisations operate based on so-called “decision premises,” which are differentiated into *decidable* and *undecidable decision premises* (47, 56). These are structural conditions that shape and guide decision-making processes.

*Decidable decision premises* refer to a university's capacity to act and decide autonomously. They are internal structures and conditions that can be determined and modified by the university itself. These include *decision programmes* (e.g., the university's mission statement), which can be differentiated into purposive (guiding decision-making by defining goals) and conditional programmes (operationalising decisions into concrete actions). These premises also encompass the allocation of roles and responsibilities among *members*, while *communication channels* form the basis for making, coordinating, and transmitting decisions across the organisation (54, 56). By actively shaping these premises, a university can respond to and adjust to both external and internal developments and perceived needs (47, 56).

*Undecidable decision premises* refer to aspects that a university cannot directly control or influence, as they are not the result of explicit organisational decisions. These premises provide an external frame for the university while also influencing internal processes and conditions. A university's *campus culture* is an undecidable decision premise that does not emerge from formal decisions, as it encompasses communicatively formed expectations, routines, and recurring patterns that can subtly yet pervasively influence the university (47, 56). Although universities primarily operate based on their internal structures and decision processes, they are inevitably in contact with and influenced by their surrounding *environment* (56), which refers not only to physical surroundings but also to external social systems (e.g., political or legal systems). According to systems theory, organisations differentiate themselves from their environment while continuously observing, filtering, and deciding if and how to react to environmental developments and demands. This leads to mutual influence (47, 56).

In summary, the systems-theoretical considerations form the foundation for the PLPU framework.

## 4 Conceptual framework of the physical literacy promoting university (PLPU)

A PLPU is defined as an *educational organisation that systematically integrates physical, cognitive, social, and affective aspects of movement-related engagement into its structures and internal decision-making processes. It aims to foster a lifelong, active and healthy lifestyle, including the development of a meaningful, autonomous, and positive relationship with movement among all its members*.

Building on the systems-theoretical considerations (46, 47, 56), a university can facilitate the systematic and sustainable integration of PL into its structures and processes by addressing the following five key components: (1) members and roles, (2) decision programmes, (3) communication channels, (4) campus culture, and (5) environmental developments and relations.

**Figure 1:**
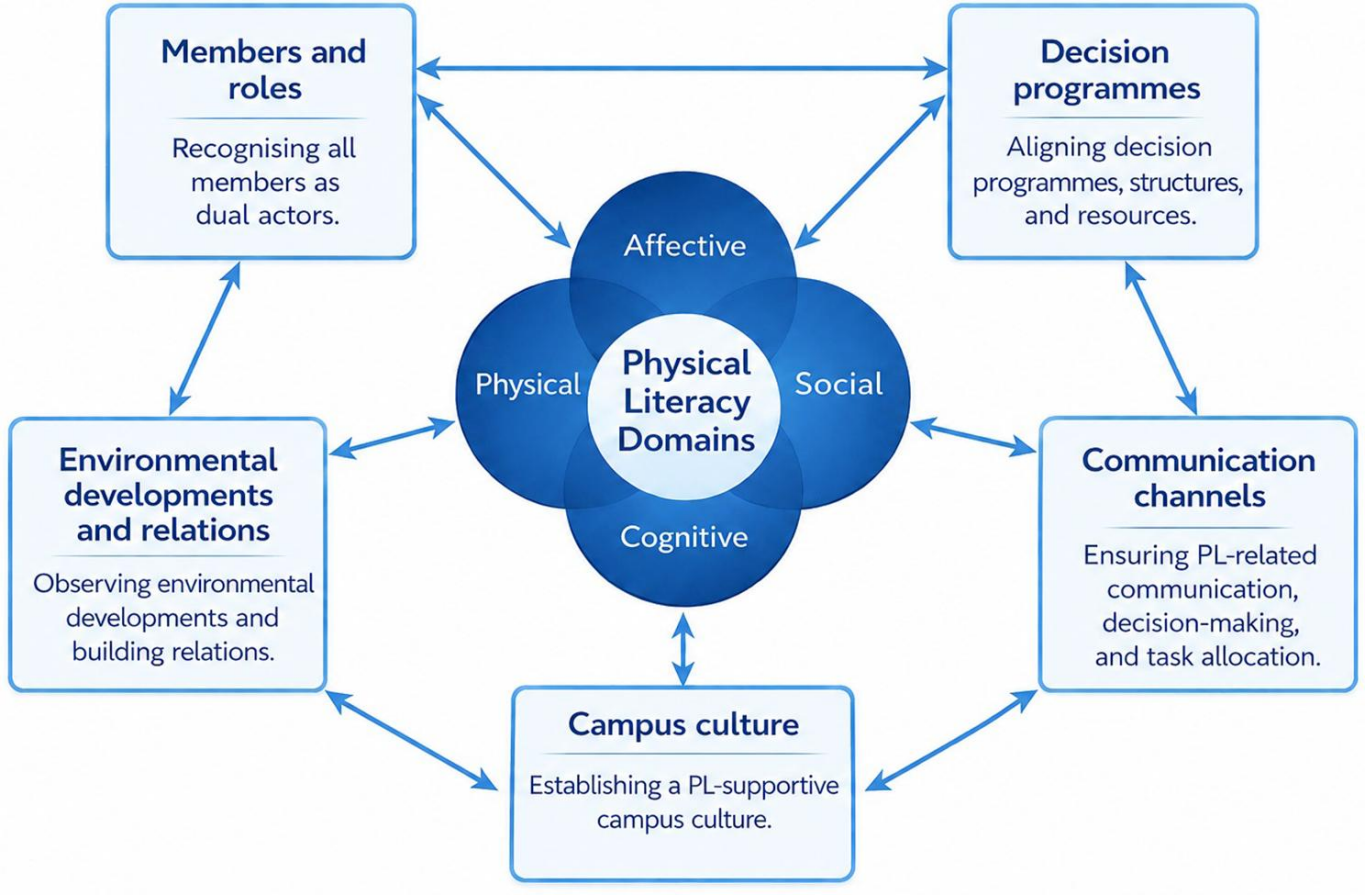
Graphical illustration of the physical literacy promoting university (PLPU) framework.

In the subsections that follow, we will outline the respective objectives and context-specific actions related to these key components (Table 1). The objectives refer to the intended outcomes of these key components within a PLPU, while the actions translate these aims into concrete, practical steps that a university can undertake (Figure 1).

**Table 1:** Conceptual framework of the physical literacy promoting university (PLPU).

Key components	Objectives	Potential actions
Members and roles	Recognising all members as dual actors in fostering and embedding PL.	Enabling members to engage with, shape, and foster PL within the university. Establishing PL-compatible human resource management.
Decision programmes	Aligning decision programmes, structures, and resources to embed and sustain PL.	Providing purposive programmes with a shared vision. Establishing PL-compatible conditional programmes and functional units. Aligning university governance structures to support PL integration. Adapting PL-supportive organisational resources and infrastructure.
Communication channels	Ensuring coherent PL-related communication, decision-making, and task allocation.	Providing PL-supportive decision-making and task allocation processes. Enhancing PL-related communication channels.
Campus culture	Establishing a PL-supportive campus culture.	Identifying PL-hindering factors within campus culture. Creating conditions that foster PL-supportive cultural development.
Environmental developments and relations	Observing environmental developments and building relations with external stakeholders.	Identifying, analysing, and acknowledging environmental PL-related developments. Establishing cooperations and networks with external stakeholders.

### 4.1 Recognising all members as dual actors in fostering and embedding PL

Focusing on university members is crucial for two main reasons. First, PL refers to a person's competences, dispositions, and lived experiences (5, 23). Second, systems theory (56) considers members as main contributors to the communication and decision-making processes through their job roles, interactions, and professional expertise (47, 56). However, university members are not a homogeneous group. They differ regarding their positions, tasks, responsibilities, and decision-making power. Therefore, it is essential to differentiate on a basic level between students, academic staff, administrative staff, and auxiliary staff, each contributing in distinct ways to promoting and embedding PL. Students may be involved through curriculum-based learning and campus activities; academic staff through the integration of PL principles into teaching or scientific work; and administrative staff through wellbeing initiatives and work organisation (41, 57). Previous PL interventions mainly focused on university students, often overlooking the role of staff members (6, 7, 58). Therefore, the PLPU framework equally addresses all members. This aligns with the PL concept, which involves all individuals regardless of their (physical) abilities, age, or competences (5, 23). Against this background, a PLPU recognises all members as dual actors: (a) individuals whose PL can be fostered, and (b) organisational actors who can actively contribute to embedding PL into the organisational structures and processes. To foster PL, a PLPU can undertake the following actions.

#### 4.1.1 Enabling members to engage with, shape, and foster PL within the university

A PLPU actively supports its members in acquiring theoretical and practical knowledge, skills and competences related to PL while raising awareness of the concept and its health-related benefits (59). This can be achieved through measures across the entire university, such as curriculum-based education programmes, movement activities on campus (7), and health management initiatives. It is crucial that members demonstrate openness to and engagement in their PL journey.

The strategic integration of participatory interventions, such as world cafés or organisational development workshops, can further enable members to actively co-create, contribute, and decide on the initiatives of a PLPU. This ensures adaptability and alignment with the diverse needs of members (60). A PLPU also encourages its members to embody and communicate the importance of PL in their regular interactions. This is achieved by enhancing a supportive climate, ensuring individual growth, and actively addressing negative behaviours such as offensive or bullying actions in PA contexts.

#### 4.1.2 Establishing PL-compatible human resource management

While it is essential to foster each member's personal PL, staff members and students also contribute to embedding PL into university structures and processes. To operationalise this dual role, PL needs to be integrated into the human resource (HR) management of a PLPU. This is the organisational unit responsible for all decisions and processes related to employees, such as recruitment, onboarding and staff development, while also cooperating with other units (55, 57, 61).

Integrating PL into HR management can create structural and cultural conditions that support initiating and shaping of PL-related measures and embedding them into the respective structures. In particular, during recruitment processes, PL can be integrated into considerations of job profiles and interviews. During the onboarding process, the HR management can introduce the university's PL measures through orientation sessions or informational materials. Within everyday operations, HR management can establish PL-supportive structures, for instance, by allocating time for employees' participation in PL workshops (56, 57).

### 4.2 Aligning decision programmes, structures, and resources to embed and sustain PL

Beyond the relevance of members, a PLPU also requires organisational structures and mechanisms to systematically plan PL-related measures and translate them into sustained actions. Therefore, *decision programmes* serve as the second key component of the PLPU framework. Within universities, a range of decision programmes already exists (e.g., mission statements, teaching and learning strategies) (41, 56, 62). However, these rarely refer to PL within the university's core areas of education, research, and administration. A PLPU integrates PL into existing programmes or establishes new PL-sensitive ones. With reference to systems theory (46, 56), establishing specific decision programmes can support a PLPU in acting autonomously and making PL-related decisions, which can encompass a broad range of potential actions.

#### 4.2.1 Providing purposive programmes with a shared vision

A central prerequisite for aligning PL with strategic decision programmes is the establishment of purposive programmes with a holistic vision of a PLPU. Such purposive programmes provide guidance for decision-making and clarify the goals of the PLPU (46, 56). Underpinned by the holistic concept of PL (5), the vision refers not only to focusing on physical competences but also to rewarding social interactions, increased knowledge and understanding of health-enhancing activities, and motivation to be active in physical contexts. Consistent communication of the vision through formal (e.g., mission statement) or informal formats (e.g., movement classes) supports the embedding of this vision into practical actions and shaping the decision programmes.

Guided by the vision, within purposive programmes, the goals of a PLPU are defined, including (a) assessing members’ needs and PL levels (63, 64), and (b) defining (sub-)goals (e.g., assigning PL as a priority in the mission statement).

#### 4.2.2 Establishing PL-compatible conditional programmes and functional units

Building on the shared vision and purposive programmes, a PLPU requires *conditional programmes* and clearly defined functional units to operationalise decision-making and translate strategic goals into practical actions. These conditional programmes are aligned with functional units, each consisting of one or several job positions with specific responsibilities and tasks (46, 56). Within a PLPU, existing units can be adapted or new dedicated PL-related units can be established to directly allocate PL-specific processes and tasks based on the goals within the purposive programmes. This ensures a clear assignment of responsibilities and PL-related tasks. A key priority could be to establish a working group on PL with shared decision-making responsibilities. This group would have the authority to plan, regularly discuss, and work towards achieving the defined goals. By embedding such functional units within the organisational structures, a PLPU ensures that decisions are actionable, responsibilities are clear, and that PL promotion is systematically implemented across the university.

#### 4.2.3 Aligning university governance structures to support PL integration

Board members and their governance structures play a central role in contributing to a PLPU. Governance groups are composed of job roles that encompass collective responsibility for central decisions across the entire university (48, 56). Therefore, a PLPU involves the existing governance structures in communication and decision-making processes, starting with the alignment of its vision and strategic decision programmes.

While governance structures have been documented in school-based PL interventions (65, 66), limited consideration exists for university contexts (30, 35). As part of the PLPU, the university's governance group is involved in the decision programmes and meets regularly with the PL working group to discuss, decide, and define (sub-)goals. By visibly showing commitment and support, board members can increase acceptance for PL promotion. Endorsement among the governance group can be enhanced through the provision of a short introduction to the PL concept, its theoretical background, and strategic purpose.

#### 4.2.4 Adapting PL-supportive organisational resources and infrastructure

To translate decision programmes and goals into practical measures and actions, organisational resources and infrastructure are essential. These refer to personnel, material and IT resources, as well as spatial facilities (47, 56). Previous PL interventions in university contexts have demonstrated that resources and infrastructure, such as sports facilities, classrooms or workshop instructors, are essential for PL promotion (6, 34).

IT infrastructure and digital resources serve as the foundation for digital dissemination and accessibility (56). For instance, platforms such as intranet or administration systems allow all members to access PL-related information or book available PL workshops. They can also provide an overview of PL-related programme schedules and guidelines or send invitations for upcoming PL discussion rounds or quantitative surveys.

A university's spatial infrastructure, such as sports facilities, courts, tracks and fields on campus, can contribute to PA behaviour. Additionally, classrooms, libraries or study rooms can facilitate active learning by providing, for instance, PL-related information and advertisement posters. Clear and strategically placed signposts across the campus can also provide navigation assistance to guide members toward sports facilities and activity zones (67).

### 4.3 Ensuring coherent PL-related communication, decision-making, and task allocation

In line with the central role of communication in systems theory (56), communication channels represent the third key component of the framework. They form the basis for decision-making, dissemination of information and verbal or written conversations among individuals across the university (46, 56, 68). Within the PLPU, communication channels facilitate actions that target the integration of PL into formal and informal communication through the establishment of clear decision-making and task allocation processes.

#### 4.3.1 Providing PL-supportive decision-making and task allocation processes

The establishment of PL-supportive decision-making and task allocation processes is fundamental within a PLPU, as these enable the dissemination of decisions and ensure smooth practical operations while specifying who does what, when, and how (47, 56). For instance, new tasks and work steps based on the decision programmes of a PLPU, such as planning PL assessments or scheduling PL-related workshops, need to be communicated and therefore integrated within existing workflows or form the basis for new ones. In alignment with the decision structures and functional units of a PLPU, responsibilities and authorities for such tasks and procedures need to be clearly assigned and communicated (56, 68). While such newly arising PL-related responsibilities and tasks may initially be unfamiliar and challenging to integrate, they can lead to more coherent processes.

#### 4.3.2 Enhancing PL-related communication channels

Communication channels are relevant for allocating and communicating PL-related decisions and tasks within a PLPU. They can take on more or less formalised forms (46, 56). Formal communications refer to structured, rule-based exchanges in reference to the decision premises that follow organisational processes and hierarchical principles (56). Within a PLPU, these formalised channels include meetings, e-mails or official documents through which PL-related announcements, tasks or responsibilities are shared and decisions are made binding. From the perspective of the systems theory, such formal communication channels do not solely serve an informational purpose; they are the central mechanism for enabling and reproducing decisions (56). For instance, documented PL-related governance group decisions or published policy statements are concrete actions that demonstrate the commitment of a PLPU by making decisions and actions visible. Ensuring that these communications refer to the decision programmes strengthens the coherence and legitimisation of decision-making (47, 56). With reference to the PL concept, formal communication channels should represent the holistic characteristics of PL, including its domains, inclusive character, and dynamic development.

Alongside such formal formats, universities also encompass less formal, spontaneous interactions among members (46). Within the PLPU, informal communication plays a pivotal role in shaping a PL-supportive, engaging climate and campus culture. Informal conversations provide members the opportunity to share impressions, opinions and PL-related experiences while enhancing greater acceptance and participation in the actions of the PLPU (47, 56). Thus, a PLPU actively creates and supports opportunities for conversations about movement experiences and exercise-related activities. For instance, peer-to-peer recommendations can foster social interaction, engagement, and participation. Additionally, informal conversations can provide bottom-up feedback through which concerns or new ideas can emerge, helping to identify needs and new approaches. To support such feedback processes, a PLPU should provide low-threshold opportunities (e.g., feedback tools or participatory workshops) to collect barriers, dissatisfaction, or proposals related to PLPU measures.

As a PLPU, it is important to note that all communication processes should follow inclusive, accessible, ethically sensitive, and non-stigmatising principles. With regard to movement-related communication, a key challenge lies in avoiding moralising or performance-oriented messages or actions which may induce guilt in inactive individuals, privilege high-performing bodies, or exclude people with disabilities or negative movement-related experiences. Instead, communication processes should be sensitive to bodily and social diversity. Following these principles, communication channels should also be accessible and adaptable to the needs and characteristics of all members.

### 4.4 Establishing a PL-supportive campus culture

The fourth key component of the framework represents the university's campus culture. Systems theory conceptualises an organisation's culture as a set of undecidable decision premises, which an organisation cannot directly control (47, 56). Campus culture is not actively decided upon; it develops through communicatively formed expectations, routines and recurring patterns that can indirectly shape the internal decisions, processes, and actions (56) of the PLPU. Within PL research in the university context, culture has not yet been explicitly addressed (6, 7, 30). However, within school-based interventions, culture has been integrated, as seen in Telford et al. (66), who reported a school's cultural shift through a PL intervention.

#### 4.4.1 Identifying PL-hindering factors within campus culture

An organisation's culture is relatively stable and slow to change (46, 56). Therefore, identifying and understanding cultural patterns, expectations and routines that can indirectly influence how decisions are made, interpreted and communicated is crucial within a PLPU. Examining potentially PL-hindering cultural factors can, for instance, be conducted through focus group discussions or workshops (60). Such hindering factors may refer to a limited understanding or ambivalent staff expectations towards PL and its measures, especially when considering the different biographical and position-related experiences of academic, administrative, and auxiliary staff members.

#### 4.4.2 Creating conditions that foster PL-supportive cultural development

While a PLPU cannot directly influence or change campus culture, it can inform and contribute to adjustments within structural, communicative, and strategic actions (56). Such mechanisms can include symbolic communication, such as the visible integration of PL into strategic documents; environmental nudges, such as providing movement-friendly spaces or prompts on campus; or communication-based approaches, such as addressing certain misconceptions or expectations regarding PL to clarify its meaning and relevance for embedding PL within the university. Although such measures cannot directly control campus culture, they can gradually contribute to a more supportive, empowering, and motivational climate that supports the fostering of PL within the university context.

### 4.5 Observing environmental developments and building relations with external stakeholders

As stated by Whitehead (26), with reference to existentialist philosophy, an individual's embodied interaction with the environment can contribute to the development of PL. However, previous PL interventions in university contexts have given relatively limited consideration to environmental factors (6, 7, 30). Systems theory refers to organisations differentiating themselves from their environment while maintaining continuous interaction by observing, reflecting and reacting to each other's developments (56, 69). This observation allows the PLPU to consider perceived environmental changes within its internal processes, structures and decision programmes (69). Therefore, *environmental developments and relations* represent the fifth key component of the framework.

#### 4.5.1 Identifying, analysing, and acknowledging environmental PL-related developments

The environment encompasses different subsystems such as political, health, education, legal, economic, and social systems (56, 69). A PLPU systematically observes and analyses environmental developments and decides if and how certain changes may inform and contribute to its structures, decision programmes and tasks (56, 69). The PLPU must also identify and analyse societal changes and demands regarding movement and sports, such as adapting advertising campaigns and communication channels from analogue to social media or making decisions regarding the incorporation of new trend activities into its programmes. Furthermore, a PLPU must be aware of and adapt to legal developments, such as changes in occupational health and safety laws, which can impact PL-related initiatives on campus. Acknowledging and responding to such environmental developments ensures that a PLPU is adaptive and aligned with external developments and demands.

#### 4.5.2 Establishing cooperations and networks with external stakeholders

In addition to observing environmental developments, a PLPU builds and engages in cooperations with external stakeholders. Such cooperations and networks can be established, for instance, with sports clubs, schools, consultants, and health insurance companies, which can offer PL-compatible courses in sports clubs and gyms outside of campus at discounted prices for university members. Exchanging knowledge regarding PL integration and promotion can be facilitated through teacher education programmes (65). Furthermore, internships with companies or communities can provide students with practical PL-related experiences. Engaging in such environmental relations establishes conditions in which environmental developments and insights can inform and contribute to the PLPU.

However, cooperations with external stakeholders are not to be understood as a substitute for embedding PL within the core structures of a university. Rather, external cooperations should complement and build on existing structures and processes while recognising the potential risks and unintended consequences they may entail, such as prioritising commercial or performance-oriented interests, creating unequal access to specific services, or fostering a narrow, fitness-oriented understanding of movement. Therefore, these cooperations require critical reflection and continuous evaluation to ensure their compatibility with the holistic principles of PL, as well as with principles of accessibility, inclusion, sustainability, and the avoidance of conflicts of interest.

## 5 Conclusion

Previous research on PL in the university context predominantly focused on individual-based approaches for enhancing engagement in movement-related activities (6, 7, 30). Accordingly, no conceptualisation exists that addresses how universities can systematically align their structures and actions to embed and promote PL at the organisational level. The goal of the present article is to introduce the conceptual framework of the PLPU by providing theoretical and practical insights into how PL can be embedded into universities through an organisational perspective.

Capitalising on an established differentiation from research on health literacy (49), while building on the systems theory (47, 56), this article introduces and differentiates between “personal PL” and “organisational PL”. The conceptualisation of organisational PL serves as the foundation for the PLPU framework. Based on the understanding of the lifelong journey of PL (5, 23), the framework considers embedding and promoting PL as an open-ended iterative process that also unfolds within universities as learning and working settings (41, 46, 48).

Practically, the framework provides strategic orientation based on its five key components, translating theoretical considerations into practical actions. Furthermore, the framework helps to align the goals of embedding and promoting PL with the university's overall objectives, including academic, educational, and administrative processes. Simultaneously, the framework encourages cross-sectoral cooperations within the university and with external stakeholders.

However, a few limitations need to be acknowledged. The PLPU framework was developed through an iterative theory-guided conceptual framework development process based on purposeful reflection and integration of different empirical as well as conceptual research findings. However, it should not be understood as the result of a systematic review, an empirical co-design process or as an empirically tested model.

The systems theoretical approach with its underlying organisational theory, also implies some blind spots. By emphasising decision-making structures and communication processes, it places less explicit focus on the role of power relations, social inequalities, leadership dynamics, and detailed implementation processes (46, 47, 56). In practical terms, the applicability and feasibility of the framework may vary depending on contextual conditions within each university, such as organisational resources, policies, campus culture, governance structures, and existing health promotion and sports infrastructure. Consequently, the framework's implementation will differ between public and private universities, resulting in varied opportunities and challenges for embedding PL within organisational structures and processes.

However, the framework provides strategic orientation for universities aiming to structurally embed PL. The implementation should follow a context-sensitive and structured pathway. This can include needs and resource assessments, the establishment of dedicated working groups, the development of a shared vision, the integration of PL into strategic and organisational documents, the piloting of PL-related initiatives, and continuous communication and evaluation strategies.

Future research should empirically assess the framework's applicability and feasibility across different university contexts. In particular, case studies and longitudinal research are needed to explore promoting and hindering factors regarding the embedding of organisational PL and the development of a PLPU over time. Therefore, potential organisational barriers should be critically reflected upon, such as organisational inertia, competing organisational priorities, limited resources, governance constraints, or insufficient stakeholder engagement. Furthermore, the framework can also guide the development of assessment tools for evaluating organisational PL and its integration within universities. In this context, future research may further operationalise the framework by incorporating organisational indicators (e.g., stakeholder participation in co-design processes, PL-related training programmes, the existence of a PL strategy, budget allocation, or the integration of PL into mission statements) to facilitate empirical assessment of its implementation across university settings.

In summary, the PLPU framework bridges the gap between individual capacities and organisational responsibilities, offering a new perspective on how universities can embed and foster PL while highlighting each university's potential to build and provide PL-enhancing structures.
